# Experiences and perceptions of social eating for patients living with and beyond head and neck cancer: a qualitative study

**DOI:** 10.1007/s00520-022-06853-6

**Published:** 2022-01-24

**Authors:** Mark Dornan, Cherith Semple, Anne Moorhead

**Affiliations:** 1 School of Nursing, Institute of Nursing and Health Research, Ulster University, Newtownabbey, UK; 2 Cancer Services, South Eastern Health and Social Care Trust, Belfast, UK; 3 School of Communication and Media, Institute of Nursing and Health Research, Ulster University, Newtownabbey, UK

**Keywords:** Head and neck cancer, Social, Eating, Commensality, Survivorship, Qualitative

## Abstract

**Purpose:**

Patients with head and neck cancer (HNC) describe eating as more than a physical activity for nutrition and calories. After treatment for HNC, patients report a changed social experience around food, with eating and drinking in front of family and friends depicted as a challenge. However, there is limited research exploring how patients with HNC adapt and cope with social eating difficulties. This study aims to explore patients’ experiences and perceptions of social eating and drinking following treatment for HNC.

**Methods:**

A qualitative research design using semi-structured interviews was employed to understand the experiences of social eating of patients living with and beyond HNC. Reflexive thematic analysis was used to inductively develop key themes from the data.

**Results:**

Fourteen interviews were conducted with patients, and two key themes were identified: (1) “Social eating became a conscious process” and (2) “Strategies to maximise social eating participation”. To maximise social eating enjoyment, patients attempted to minimise the attention on their eating function and the fuss created around food. Patients with HNC established psychological and cognitive adaptations to manage expectations and promote positive participation in social eating.

**Conclusion:**

This paper identifies key barriers limiting or diminishing social eating for patients with HNC; including being self-conscious, lack of understanding from others and functional issues with eating and drinking. This research highlights the need to raise awareness of social eating challenges and for the social dimensions of eating to be addressed through family-centred, supportive holistic interventions implemented early in the patient’s cancer journey.

## Introduction

Patients who undergo head and neck cancer (HNC) treatment report substantial changes in their physical, functional and psychosocial well-being [1]. Side effects of treatment include dysphagia and other eating-related difficulties due to pain, xerostomia, mucositis and lack of appetite [2]. Ninety percent of patients with HNC have eating and drinking difficulties after treatment [3, 4]. Subsequently, food has a changed meaning, where eating and drinking with others are identified as a challenge [5, 6]. Research demonstrates that social eating is a significant problem, often continuing beyond 5 years after treatment [7]. Social eating and drinking are defined as eating or drinking in the presence of another person [8]. In this study, the phrase, social eating, is used to encompass both activities of social eating and drinking.

Our systematic review indicated that following HNC treatment, patients experienced a range of losses associated with eating and drinking socially [9]. Due to the functional side effects of treatment for HNC, for example, nasal leakage or oral incontinence, patients reported feeling self-conscious, shame and embarrassment eating in front of others [5, 10, 11]. In addition, patients chose to eat separately or only with close relatives and experienced a loss of togetherness with their wider social network and between intimate relationships [5]. Therefore, patients with social eating challenges are at risk of isolation, loneliness and diminished quality of life [12, 13]. This study is underpinned by Engel’s biopsychosocial model of health [14]; as patient’s post-treatment social eating exeperince intrinsically links physical, psychological and social dimensions. Previous research notes that post-treatment HNC challenges are often not, but should be regarded within a biopsychosocial framework [15].

Despite acknowledgement within the research that social eating is an ongoing problem for many patients, there is a paucity of literature exploring how patients cope beyond the physical and functional alterations to food and eating habits [9]. Furthermore, results from a longitudinal analysis of social eating outcomes of 5000 patients with HNC indicated a requirement for additional research to understand the needs of those at a higher risk of social eating difficulties [16]. There is a dearth of studies exploring patients’ experience and support needs to help promote social eating following treatment for HNC, which this study aims to address [17, 18]. This study aimed to explore patients’ experiences and perceptions of social eating following treatment for HNC. The objectives of the study were to:

- Explore and understand barriers that inhibit or diminish participation for patients in social eating following treatment for HNC.
- Explore the psychosocial implications of altered social eating for patients following treatment for HNC.
- Explore coping strategies to promote participation in social eating for patients following treatment for HNC, including those beyond physical and practical adjustments.

## Methods

### Design

A descriptive qualitative research design using semi-structured interviews was employed to understand the experiences and perceptions of social eating for patients living with and beyond HNC. The study is reported following the Consolidated Criteria for Reporting Qualitative Research (COREQ) guidelines [19].

### Sampling and sample

A purposive sample of patients living with and beyond HNC participated in this study. Inclusion and exclusion criteria were established, displayed in Table 1. One local collaborator, a Clinical Nurse Specialist (CNS) in HNC, identified participants from one hospital in the UK and sought permission for the researcher (MD) to contact interested participants to answer any study-related questions and arrange an interview for those willing to participate. Informed written consent was obtained.

**Table 1:** Inclusion and exclusion criteria of the patients

Inclusion criteria	Exclusion criteria
•Over the age of 18 years at the time of the interview •Completed treatment (surgery, radiotherapy, chemotherapy or combination) with curative intent for HNC •Be able to provide informed consent •Communicate in English	•Receiving ongoing treatment for recurrence, palliative or end-of-life treatment •Treatment was not yet complete

### Data collection

**Fig. 1:**
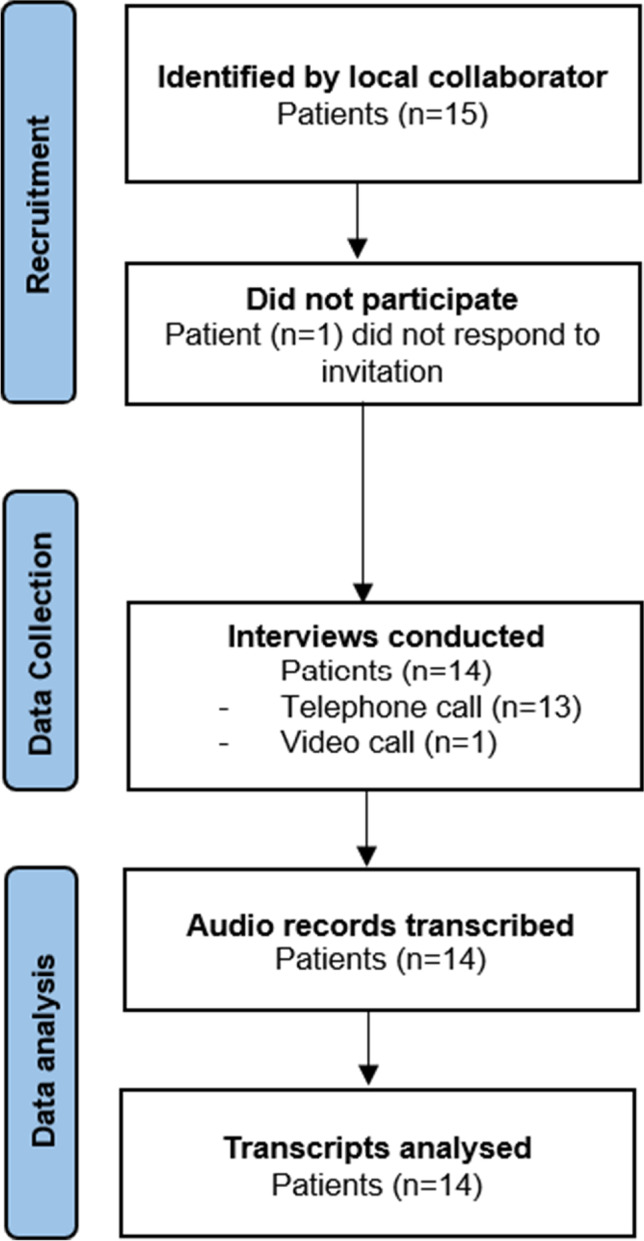
Data collection flow diagram

A topic guide was created, informed by literature and knowledge from subject experts. This was iteratively modified to reflect the research objective (Appendix). Two pilot interviews were conducted with patients with HNC to verify the content and meaning of the topic guide. Individual, semi-structured interviews were conducted from February 2021 to June 2021 via telephone or video call, due to COVID-19 restrictions. Recent research indicates that telephone or remote interviews did not negatively impact the richness of collected data [20, 21]. The first author, a Registered Nurse and academic researcher (MD), completed research training in qualitative data collection and analysis training before conduction all interviews. MD was not involved in caring for this participant population. Other research team members (CJS & AM) have extensive qualitative research experience. The duration of the interviews ranged from 24 to 53 min and was audio-recorded (Fig. 1).

### Data analysis

Each interview recording was transcribed verbatim by the researcher (MD), ensuring familiarity. The transcription was checked for validity by the research team (CJS, AM). Braun and Clark’s six-step approach to reflexive thematic analysis of qualitative data was used [22, 23]. Initially, the first author (MD) read and reread the transcripts to understand each participant’s story. Next, the transcripts were coded by the first author (MD) in NVivo V12. As an inductive process, codes were developed and collated by the first author (MD), identifying initial themes. The codes and themes were independently analysed by two authors (CJS, AM) to ensure rigour and credibility. Through iterative team discussions (MD, CJS, AM), the themes were refined, and final themes were established to ensure the correct meaning of the participants had been captured.

### Ethical considerations

This study received approval from the NHS Wales Research Ethics Committee (20/WA/0253) and research governance from local participating healthcare trusts within the UK. Due to the nature of the topic and participant group, a distress protocol was created to ensure appropriate actions or signposting was provided, if appropriate, and subsequently required for one participant. Enabling ongoing reflexive practice, whilst promoting transparency and trustworthiness, field notes were taken during the interview, reflective notes made after interviews, and regular discussions with the research team to assess one’s potential influencing role in data collection and analysis.

## Results

In total, 15 potential participants were initially identified, with 6 males and 8 females (*n* = 14) subsequently taking part in an interview (Table 2). Patients mostly lived with others (*n* = 11), were between 1 and 5 years post-treatment (*n* = 11), all having cancer of the oral cavity, with their primary treatment modality being surgery.

**Table 2:** Characteristics of the patients (*n* = 14)

Patient ID	Gender	Age (years)	Tumour location	Treatment	Time since completion of treatment	Employment	Lives with
1	M	46–60	Oral cavity	Surgery & RT	2–5 years	Full time	Spouse & children
2	F	46–60	Oral cavity	Surgery, RT, CT	1–2 years	Retired	Partner
3	F	76 +	Oral cavity	Surgery & RT	2–5 years	Retired	Alone
4	M	61–75	Oral cavity	Surgery	2–5 years	Retired	Spouse
5	M	61–75	Oral cavity	Surgery, RT, CT	1–2 years	Retired	Child
6	F	61–75	Oral cavity	Surgery & RT	2–5 years	Retired	Alone
7	M	61–75	Oral cavity	Surgery & RT	3–6 months	Retired	Spouse & children
8	F	46–60	Oral cavity	Surgery	2–5 years	Full time	Spouse & children
9	F	76 +	Oral cavity	Surgery	1–2 years	Retired	Alone
10	F	18–30	Oral cavity	Surgery & RT	5 +	Part time	Spouse
11	F	76 +	Oral cavity	Surgery	2–5 years	Retired	Spouse
12	M	46–60	Oral cavity	Surgery, RT, CT	1–2 years	Retired	Spouse & children
13	F	61–75	Oral cavity	Surgery & RT	2–5 years	Sick leave	Partner
14	M	46–60	Oral cavity	Surgery, RT, CT	3–6 months	Sick leave	Spouse & children

*RT*, radiotherapy; *CT*, chemotherapy; *M*, male; *F*, female.

Two key themes were identified following thematic analysis: (1) “Social eating became a conscious process” and (2) “Strategies to maximise social eating participation”.

### Key theme 1: Social eating became a conscious process

Patients described eating with others after HNC treatment as becoming a conscious process. The conscious process characterised some of the meaning that influenced their behaviour, attitudes and psychological challenges connected to social eating. Two distinct areas contributed to the social eating experience becoming a conscious process as demonstrated by the following subthemes: (1) “Being conscious of food” and (2) “Being conscious of how their eating appeared to others”.

### Being conscious of food

For some patients, the most significant barrier inhibiting social eating was functionality, not being physically able to eat the same food that others were consuming around them. Watching others eat food that they could no longer enjoy diminished their social experience and, on occasions, contributed to patients removing themselves from the social setting. In addition, for some patients having to alter their food, such as texture modification, deterred participation in social eating, within and outside the home.

I do find it difficult to sit watching people enjoying their pizza and enjoying their Chinese and enjoying their steak burger and whatever, I’m thinking to myself […] I’ll never eat anything like that again. (Patient 1).

After treatment, choosing and consuming certain foods became a more conscious experience when others were present. This was more apparent in other people’s homes and restaurants, where sometimes the availability of suitable food was an obstacle. In addition, communicating their needs around food to restaurant staff was often a barrier, as patients encountered a lack of understanding of their situation and requirements, including texture modification or additional sauces and gravy.

I’d say just very anxious about going out, especially with friends and things going out for dinner and being afraid of nothing, you know, there’s nothing on the menu that you think I can eat easily without anyone noticing. (Patient 10).

Many patients were conscious they now ate slowly, and when having to participate in a concurrent conversation, their eating pace was further delayed. Occasionally, this attracted attention, with a heightened focus as they were the only remaining person eating.

I know I’ve been to a restaurant with my family before and they had all finished but then the waiters and waitresses came and cleared all their plates and left mine, I was really sort of annoyed at that. (Patient 10).

After treatment, patients became conscious of food in a social context, however, this was often intrinsically linked to also being conscious of how their eating appeared to other people.

### Being conscious of how their eating appeared to others

After treatment, some patients were conscious of how their eating appeared to others and described their altered eating function as “childlike” due to mess created when eating. Patients now became conscious of how they appeared when they were eating in front of others. They were self-conscious of dribbling on their clothes and food on their face, glasses or hair. In general, patients reported they found eating with others stressful.

I have to eat with my hands, soft foods, like fish I can call into small pieces. I tried eating with a fork and I stabbed myself on the gum, which isn’t good so I eat with my hands and shove it in the three or four mil gap that I have […] there’s a confidence thing but also here you can’t open your mouth properly to eat, you definitely don’t want to be doing it in public you know so, I wouldn’t have been going out to eat anyway you know. (Patient 12).

Some patients also reported being conscious of how they ate in front of children, not wanting children to acquire some of their new eating habits which were perceived as socially unacceptable. In addition, it was recognised how younger children sometimes commented on patient’s eating habits, making them feel self-conscious.

There were like ‘why is [patient 2] not having that and you know, just silly little things that I can see in their wee faces. (Patient 2).

A minority of patients stated they did not have the confidence or functional ability to participate in any circumstances that involved social eating. However, whilst eating in front of others became a conscious process, most patients developed strategies to have some social interaction around food.

### Key theme 2: Strategies to maximise social eating participation

Alongside the various experiences and perceptions of eating with others, patients expressed some of the strategies they employed to maximise their participation in social eating, including (1) “Minimising attention on eating”, (2) “Managing expectations” and (3) “Receiving support from others”.

### Minimising attention on eating

To maximise participation in social eating, sometimes patients endeavoured to minimise attention on eating when socialising with others. Many patients wanted to minimise fuss and attention around social eating and blend in, thus avoiding scrutiny associated with their functional eating challenges or food alterations.

If they would just act normal and leave me to, you know, do my own thing. (Patient 13).

An array of methods were employed to divert focus from themselves and their food, thus redirect attention. To minimise attention on eating, some patients would not eat anything socially and eat privately or beforehand at home. Alternatively, patients might order soup, custard or coffee that may not have been as appealing but was more manageable and subsequently minimise attention on their eating ability. For some patients, eating with a group of people was less intense than eating with only one other person as they could reduce the focus on themself. However, some patients considered there was more pressure eating with strangers, for example, at a wedding. More often, patients were motivated to participate in some measure, as they enjoyed the interactions from being present.

If we were just going for, to say hello and have a drink, I would probably go, but if it was a case of going, for a meal, no, I would make sure I had food in me, but I wouldn’t go and sit down and eat you know. (Patient 12).

To reduce any potential distress around eating, some patients declined entirely to socialise around food. Instead, they might have suggested an alternative activity such as going for a walk, shopping or having a social drink, which was viewed as potentially more feasible.

### Managing expectations

To maximise social eating participation, patients had to manage their expectations of themselves, others and recovery. Patients did not anticipate the severity or chronicity that eating challenges would pose on socialising. Managing their expectations of future social eating participation involved learning to self-manage, including a trial-and-error approach to discovering what food they could eat socially. Alternatively, patients tried graded exposure or “small steps” to practice eating with close relatives before eating in a larger group or restaurants.

There was problems in the kitchen and whatever, so that was a bit disappointing, so the dinner for that night with me was pretty limited. So those are the sort of difficulties you come up against. Now that was a bad experience, if I was to approach that again, I’m sure it wouldn’t be the same. (Patient 14).

Patients were conscious of managing the expectations of their family around social eating. Family members, when endeavouring to provide support and encouragement, occasionally added additional, unwanted pressure. At times, patients considered their family’s lack of understanding surrounding their needs as barriers to a positive social eating experience.

My sister […] hadn’t put a plate down for me […] so I was huffing and puffing about that, you know, I didn’t know that she did, I wasn’t eating at that point. […] I’m surprised at her she didn’t hand me one as normal, because in any situation that’s what she normally does. (Patient 6).

Some family members wanted to continue as usual without recognising the challenges that eating with others could pose. This was frustrating for patients and potentially contributed to further exclusion. Accompanying this, patients were mindful of how their expectations and treatment impacted their friendships or family time at special events and meals around the table.

### Receiving support from others

One of the fundamental strategies promoting enjoyment with social eating was support from other people, crucially family members. Some family and friends would aim to reduce the emotional and psychological burdens that accompany social eating. They would do this by cooking food that patients enjoy and provide opportunities to socialise, not involving food. In addition, bringing immediate family to larger social meals reduced the apprehension of social eating, where family could speak to staff in restaurants on their behalf.

I’ve been out to a restaurant where my son, well over four of us, two friends and one of my sons and he had spoken to the chef and asked him could he do a steak for me but have it blended. (Patient 3).

To a lesser extent, friends provided some support; however, social eating was infrequently discussed with friends or colleagues.

I just didn’t ever bring it up, I never felt comfortable bringing it up because it was such a rare thing for anyone to have and I don’t like, yea, I just felt a bit weird. (Patient 10).

Alongside support from family, a range of HCPs was included as providing support, including their CNS, Speech and Language Therapist (SLT), Dietician and Surgeon. There was often a focus on physical attributes of food and limited conversation around the social challenges of eating. Patients reported receiving no specific information, and more general self-management techniques were encouraged.

she [CNS] would have rang up every so often and asked how things was going and tell her my problems […] And she more or less said to me, take it day by day and take it nice and easy and every day you’ll get a wee bit stronger and she was right. (Patient 4).

Finally, other patients who were living with HNC were essential contributors to peer support. This may have been chance meetings during treatment or formally organised by HCPs. However, people found a sense of understanding that they could only receive from peers, which made them feel less alone. Moreover, one respondent suggested they would be more likely to eat in front of other people with HNC as they would share a common understanding. To overcome barriers to positive social eating experiences, patients indicated a range of psychosocial coping strategies and key support providers to promote participation.

## Discussion

The novel finding this research has identified is that social eating becomes a conscious process for patients after HNC treatment, influencing patients’ behaviours, attitudes and feelings connected to social eating. Patients indicated four key barriers to social eating participation: (1) the inability to eat certain foods, (2) necessary texture modifications, (3) perceptions of how they appeared to others and (4) lack of understanding among family, friends and restaurants. This study also highlights that patients with HNC do not use physical or practical coping mechanisms alone but psychological and cognitive adaptations to promote positive social eating.

Underpinning this discussion are the sociological beliefs that eating with others, or commensality, is superior to eating alone and has a more positive effect on life satisfaction [24]. Patients in this study consistently viewed social eating as an activity that should provide pleasure, and positive participation was sought. However, as a consequence of HNC treatment, patients often perceived they could not uphold cultural expectations of acceptable behaviours, colloquially known as “table manners”, due to their functional challenges, making social eating frustrating and stressful [25].

Sociological literature identifies that familial commensality is linked to gender and family roles [26, 27]. Within this study’s cohort, being a grandparent or a parent of children (irrespective of their age), appeared to be both a pivotal motivating factor and a source of emotional support. Being a parent or grandparent prompted participation in family social eating events. From this group of patients, there was no delineation of how their gender was uniquely linked to their role of social eating within the family network, with both men and women describing their individual experiences of eating with their family. However, Patterson et al. (2021) found that women with HNC may have poorer social eating outcomes [16]. This has compelling ramifications for research and clinical practice, as the changing demographic of this patient population indicates an increase in younger women with HNC, who would therefore be at risk of diminished quality of social eating and quality of life [28].

Social eating interactions have an important role in the development of children and young adults [29]. As parents, patients were mindful of their roles and actions at the dinner table. Whilst wanting to be present for the social interaction, they were mindful of how their children may mimic their eating habits. Role and identity should be considered when addressing support needs of social eating, as social eating appeared to have more meaning for younger patients and those in employment. Patients were more attentive to social eating when it formed part of the working and social lives before HNC treatment. After treatment, patients reported that social eating required continuous motivation and organisation, which reduced the spontaneity of going out for meals, taking trips away or trying new or different food [4]. Other HNC literature demonstrates that previous life experiences influence how patients cope with psychosocial challenges associated with treatment and survivorship [30].

Family members are regarded as the most essential support providers for social eating [9]. Patients reported a precarious balance between supportive encouragement and undue pressure with social eating from relatives, sometimes created tension within family relationships. This was more evident if patients considered family members compelled their attendance at social eating environments. However, patients reflected that sometimes their challenges with social eating impacted their family’s enjoyment of eating. Therefore, balancing and supporting family members’ emotional and social needs is an additional aspect to consider within cancer survivorship [31].

Consequences of HNC treatment, specifically those impacting eating and drinking, can be longstanding [32, 33]. This study identified that some patients delayed eating in front of others; anticipating their ability to eat in a socially acceptable manner would return. However, some patients established a routine of eating alone and contemplating future participation in social eating made them feel anxious. Thus, due to the chronicity of HNC treatment-related side effects which impact psychosocial well-being, early supportive social eating interventions are required [34].

There is a growing interest in survivorship research regarding the role of “prehabilitation” to reduce the severity and impact of treatment and maximise long-term function through early interventions [35]. Initially, the focus was promoting physical function alone but prehabilitation has extended to incorporate psychosocial strategies [36]. However, there is no consensus on optimal timing for prehabilitation [37]. Within this study, patients differed in their opinion on the timeliness of a social eating intervention. A dichotomy between pre-treatment and initially post-treatment may indicate that the delivery of a supportive social eating intervention should not be seen as a one-time event but require monitoring and continuous support as the patient’s goals, preferences and functional change from diagnosis to ongoing survivorship.

### Study limitations

All patients participating in this research had oral cancer, surgery as primary treatment and none solely tube-dependent; therefore, they may not represent all HNC patients. Furthermore, due to COVID-19 pandemic restrictions, this study was conducted remotely rather than face-to-face. It appears that some patients preferred this as it minimised disruption to daily life, and research invitation uptake was high. In addition, some participants had not eaten with others, apart from close family, or in restaurants in many months since completing treatment due to pandemic restrictions. Thus, people were having to think about their retrospective experiences or additionally, people who had more recently completed HNC treatment were viewing the potential and prospective ideas of eating in front of others.

### Clinical implications

Consistent with existing research, support from HCPs was mainly limited to physical and functional or “medicalised” nature of food [38]. Advice provided centred on swallowing support from SLTs and calorie-rich supplements from Dieticians. This research demonstrates an evident need to raise awareness of the biopsychosocial aspects of eating among HCPs, alongside hospitality staff regarding ongoing challenges and implications of altered eating for patients with HNC and their families. Recent results from the HNC5000 study, which paid particular attention to social eating, recommended developing interventions to address the social consequences of dysphagia [16]. However, extant interventions developed in this area have limited generalisability to everyday life or routine clinical care [17, 39, 40]. Thus, the need for a supportive intervention to be developed that is translatable and achievable in a real-life context is paramount.

### Future research

Additional research is required to explore the role of social eating across geographical regions, demographies and other areas of healthcare with patients who have altered eating requirements due to medical or health needs. Given the impact social eating has on the lives of family members, any future interventions should be family-centred and provide information to primary support providers [41, 42]. Future interventions must support patients to overcome challenges and tackle barriers they experienced in restaurants and their social network due to a lack of understanding and limited available information.

## Conclusion

This research identifies key barriers that diminish or limit enjoyment in social eating for patients who have had treatment for HNC, such as being self-conscious, lack of understanding from others and functional issues with eating and drinking. The role of family, particularly being a parent or grandparent of young children, has motivational influences on social eating. This research highlights the need for family-centred, supportive holistic interventions to be implemented early in the patient’s cancer journey. The social dimension of eating must be addressed through ongoing holistic assessments, raising awareness of HNC survivorship needs and supportive interventions for patients living with and beyond HNC and their families.

## Appendix Topic guide for interview

### Eating and drinking challenges following treatment

- What was your experience of eating with others after treatment?
- Was that different at home/work/out of house socially?
- What about **celebrations**/events such as weddings/birthdays/BBQs etc., is that different now?
- Has your ability to eat and drink **changed over time**, since you finished treatment?
- What/who has helped your ability to eat and drink over time?
- How has eating and drinking with others changed over time since your treatment? Is it better/more difficult?

### Relationships

- Describe how or if any changes to your eating and drinking patterns has impacted on any relationships (friends/family/partner/work colleagues)?
- Is there anything that your friends/family/partner/colleagues done that has been improved or facilitated your ability to eat or drink with others?
- Did your friends or family do anything that was good or helped eating together?

### Coping

- Explore key barriers to eating and drinking socially following treatment.
- Is there anything that has helped you cope with social eating and drinking difficulties? (Probe: Physically, emotionally, psychologically, socially)?
- What do you do that helps you eating and drinking in front of others?
- Could you describe any strategies that you found helpful to improve social eating?
- Explore strategies used to improve social eating – (Probes: trial and error, graded exposure, eating at certain times/places/people/food)?
- What strategies have you found helpful?
- Anything that has been unhelpful?

### Support

- Have you received any help or support about eating socially? (Probes: from family/friends/HCPs)?
- Who has been helpful? What has been helpful? What has not been useful to you?
- What support would you like, or do you think would be beneficial?

### Intervention

- What information do you think we should it contain?
- When would be a good time for you to hear about this support? Before/during/after treatment?

## Data Availability

N/A.
